# Campbell title registrations to date – November 2022

**DOI:** 10.1002/cl2.1292

**Published:** 2022-12-13

Details of new titles for systematic reviews or evidence and gap maps that have been accepted by the Editor of a Campbell Coordinating Group are published in each issue of the journal. If you would like to receive a copy of the approved title registration form, please send an email to the Managing Editor of the relevant Coordinating Group.

## AGEING

Non‐pharmacological interventions for older depressed people: An evidence and gap map

Wenru Shang, Yanfei Li, Lili Wei, Yujia Liu, JunQiang Niu, Xiuxia Li, Kehu Yang

25 October 2022

## BUSINESS & MANAGEMENT

Employee work motivation, effort and performance under a merit pay system: A systematic review

Cédric Velghe, Pinar Celik, Stan De Spiegelaere, Anders McIlquham‐Schmidt, Martin Storme

7 November 2022

## CRIME & JUSTICE

Trauma‐informed care in carceral occupational contexts

Denise M. Brend, Zack Marshall, Élyse Granger

9 November 2022

Correlates and antecedents of neighbourhood hate crime: A systematic review of risk and protective factors

Kathryn Benier, Michelle Sydes, Angela Higginson

4 November 2022

## CLIMATE SOLUTIONS

Carbon pricing and health; impact and opportunities for transformative change: an evidence map, systematic review and framework analysis

Soledad Cuevas, Daniel Nachtigall, Aimee Aguilar Jaber, Kristine Belesova, Jane Falconer, Andy Haines, Tamzin Reynolds, Hugh Waddington, Rosemary Green

7 October 2022

## DISABILITY

Compilation and mapping of functional assessment tools to the ICF‐CY brief core set for children and adolescents with ADHD: A scoping review

Madhavilatha Maganti, Meenakshi Girish, Shashikala Gopalaswamy, Santanu Tripathi, Sujiv Akkilagunta, Sunanda Kolli, Kawaljit Multani, Maria Lewin, Shilpa Sridharamurthy, Dinesh Saroj, Sofia Azad, Hamsalekha M C, Niharika Vellakkatt, Monika Bhalvani

17 October 2022

Psychological factors as predictors for persistent post‐concussion clinical outcomes: an integrated literature review protocol

Katherine Buzzanca, Jason Beneciuk, Jane Morgan‐Daniel, Aliyah Snyder, Russell Bauer, Sarah Lahey, Russell Addeo, Zachary Houck, Christopher Perez

9 August 2022

## EDUCATION

Assessing the use of the Population, Interventions, Comparator and Outcomes (PICO) framework in studies published in education: A meta‐survey

Jiří Kantor, Roisin P. Corcoran, Jian Du, Jiaoli Li, Danping Peng, Monika Ptáčková, Dagmar Sedláčková, Alžběta Smrčková, Kateřina Veselá, Shulan Zeng, Holger J. Schünemann

7 November 2022

The effectiveness of teaching science experimentation using virtual reality for primary and secondary school students: A systematic review

Xiaoling Hu, Lingxia Zhao, Liping Guo, Mingfang Li and Kehu Yang

4 October 2022

The effectiveness of sexuality education on adolescent sexual knowledge and behaviour: a systematic review

Xinyu Huang, Chunyan Liu, Zhipeng Wei, Qin Yang, Howard White, Liping Guo, Kehu Yang

2 September 2022

School‐based screening to capture unmet social and emotional needs in children and young people: A scoping systematic review of the Boxall Profile

Angeliki Kallitsoglou, Antonia Simon, Olympia Palikara

2 September 2022

Education interventions for the teaching and learning of evidence based practice in developing countries: A systematic review of undergraduate healthcare students

Dorothy I. Nalweyiso, Josette Bettany‐Saltikov, Joseph Kabanda, Alison A. Kinengyere, Aloysius G. Mubuuke, Katherine Sanderson

25 August 2022

An evidence and gap map: Immersive reality in higher education during the Covid‐19 pandemic

Vickel Narayan, Thomas Cochrane, James Birt

25 August 2022

Information literacy education for improving health information‐seeking‐behaviour in Black American communities: A systematic review

Bethany McGowan, Jahala Simuel, Christina Harrington

24 August 2022

Effectiveness of sexual and reproductive health blended learning approaches for capacity strengthening of health professionals in low‐ and middle‐income countries: A systematic review

Elizabeth A. Kumah, Florence Mgawadere, Alice Ladur, Sarah White, Zainab Suleiman, Nicholas Furtado, Charles Ameh

16 August 2022

